# Knockout of CAFFEOYL-COA 3-O-METHYLTRANSFERASE 6/6L enhances the S/G ratio of lignin monomers and disease resistance in *Nicotiana tabacum*

**DOI:** 10.3389/fpls.2023.1216702

**Published:** 2023-10-05

**Authors:** Mingxin Liu, Huayin Liu, Jianduo Zhang, Cui Li, Yinke Li, Guangyu Yang, Tong Xia, Haitao Huang, Yong Xu, Weisong Kong, Bingzhu Hou, Xiaoquan Qi, Jin Wang

**Affiliations:** ^1^ Research and Development of Center, China Tobacco Yunnan Industrial Co., Ltd., Kunming, China; ^2^ School of Ethnic Medicine, Yunnan Minzu University, Kunming, China; ^3^ Technology Center, China Tobacco Yunnan Industrial Co., Ltd., Kunming, China; ^4^ Key Laboratory of Plant Molecular Physiology, Institute of Botany, Chinese Academy of Sciences, Beijing, China; ^5^ University of Chinese Academy of Sciences, Beijing, China

**Keywords:** CCoAOMT 6/6L, lignin, tobacco bacterial wilt, tobacco brown spot, transcriptome, metabolome, soyasaponin Bb, *Nicotiana tabacum*

## Abstract

**Background:**

*Nicotiana tabacum* is an important economic crop, which is widely planted in the world. Lignin is very important for maintaining the physiological and stress-resistant functions of tobacco. However, higher lignin content will produce lignin gas, which is not conducive to the formation of tobacco quality. To date, how to precisely fine-tune lignin content or composition remains unclear.

**Results:**

Here, we annotated and screened 14 *CCoAOMTs* in *Nicotiana tabacum* and obtained homozygous double mutants of *CCoAOMT6* and *CCoAOMT6L* through CRSIPR/Cas9 technology. The phenotype showed that the double mutants have better growth than the wild type whereas the S/G ratio increased and the total sugar decreased. Resistance against the pathogen test and the extract inhibition test showed that the transgenic tobacco has stronger resistance to tobacco bacterial wilt and brown spot disease, which are infected by *Ralstonia solanacearum* and *Alternaria alternata*, respectively. The combined analysis of metabolome and transcriptome in the leaves and roots suggested that the changes of phenylpropane and terpene metabolism are mainly responsible for these phenotypes. Furthermore, the molecular docking indicated that the upregulated metabolites, such as soyasaponin Bb, improve the disease resistance due to highly stable binding with tyrosyl-tRNA synthetase targets in *Ralstonia solanacearum* and *Alternaria alternata*.

**Conclusions:**

CAFFEOYL-COA 3-O-METHYLTRANSFERASE 6/6L can regulate the S/G ratio of lignin monomers and may affect tobacco bacterial wilt and brown spot disease resistance by disturbing phenylpropane and terpene metabolism in leaves and roots of *Nicotiana tabacum*, such as soyasaponin Bb.

## Introduction

*Nicotiana tabacum* is an important economic crop, which is planted worldwide. Tobacco production involves many processes such as planting and baking, among which resistance, growth, baking ability, quality, and other indicators are key traits for evaluating excellent varieties. In the field, the bacterial wilt and brown spot are bacterial and fungal diseases caused by the infection of roots and leaves by *Ralstonia solanacearum* and *Alternaria alternata*, respectively, which are the most serious threats to the quality of tobacco leaves (Tong et al., 2012; Lan et al., 2014). The application of resistant varieties is one of the effective approaches for disease control and key trait improvement, and these are also closely related to metabolism during tobacco cultivation (Lebeau et al., 2011).

Lignin is a kind of phenolic macromolecular polymer in plants, which is the main component of the secondary cell wall. It widely exists in tobacco and has important physiological functions. For example (1), it enhances the physical and mechanical properties of plants and improves the strength of stems (Jung and Ni, 1998); (2) it is conducive to the transportation of water and nutrients and improves the water retention capacity of plants (Bernard-Vailhé et al., 1996; Hopkins et al., 2001; Pedersen et al., 2005; Webster et al., 2005); and (3) it provides defense against biological invasion such as pathogens and nematodes, as well as abiotic stresses such as drought and low temperature (Pellegrini et al., 1993; Martz et al., 1998; Maury et al., 1999; Guillaumie et al., 2009). Generally, lowering the lignin content will cause abnormal growth or even death of plants, so it plays an important role in maintaining normal growth and development of plants. However, higher lignin content will produce lignin gas, which is not conducive to the formation of tobacco quality (Li et al., 2011; Zang et al., 2015; Jin et al., 2018). Therefore, the precise fine-tuning lignin content or composition will become a breakthrough to explore better-performance tobacco varieties.

Lignin is composed of three kinds of monomers, namely, guaiacyl lignin (G-monomer), syringyl lignin (S-monomer), and hydroxy-phenyl lignin (H-monomer). Its biosynthesis includes monomer synthesis and monomer polymerization (Rastogi and Dwivedi, 2008; Li et al., 2010; Vanholme et al., 2010). The biosynthesis of lignin monomer needs to go through the shikimic acid pathway, the phenylpropanoid pathway, and the specific lignin pathway in turn. Phenylalanine undergoes deamination reaction, hydroxylation, methylation reaction, redox reaction, and a series of reactions to finally produce three monomers (Li et al., 2010; Vanholme et al., 2010). Caffeoyl-CoA 3-*O*-methyltransferase (CCoAOMT) and caffeic acid 3-*O*-methyltransferase (COMT) are two key enzymes in the specific lignin pathway (Xie et al., 2019).

CCoAOMT can catalyze the phenol hydroxymethylation at the C3 position on the benzene ring of caffeoyl-CoA, that is, the formation of the G-monomer; COMT can catalyze C3 and C5 positions of the benzene ring at the same time, that is, the S-monomer (Li et al., 2010). The free C-5 position on the G-monomer can react with other monomers to form a stable C–C cross link, which is relatively difficult to separate and degrade, whereas the S-monomer is relatively easy to degrade, so the S/G ratio is often used as an important indicator to evaluate lignin (Jung and Ni, 1998; Christensen et al., 2000; Rastogi and Dwivedi, 2008; Verma and Dwivedi, 2014). In *Arabidopsis*, the mutation of *AtCCoAOMT1* does not affect the growth and development of plants but can reduce the biosynthesis of G-monomers and thus increase the S/G ratio (Xie et al., 2019); however, the double mutant *comtccomt* will cause abnormal growth and development, leading to death (Do et al., 2007; Nelson et al., 2017).

In this study, we annotated and screened *CCoAOMTs* in *Nicotiana tabacum* and obtained homozygous double mutants of *CCoAOMT6* and *CCoAOMT6L* through CRSIPR/Cas9 technology. It was found that the double mutants have better growth and disease resistance than the wild type whereas the S/G ratio increased and the total sugar decreased. Furthermore, through the multi-omics and molecular docking, we considered that S lignin and upregulated compounds, such as soyasaponin Bb, may enhance tobacco growth and resistance. *CCoAOMT6/6L* may be the potential genes for tobacco breeding.

## Results

### Identification of the *CCoAOMT* gene family in tobacco

**Figure 1:**
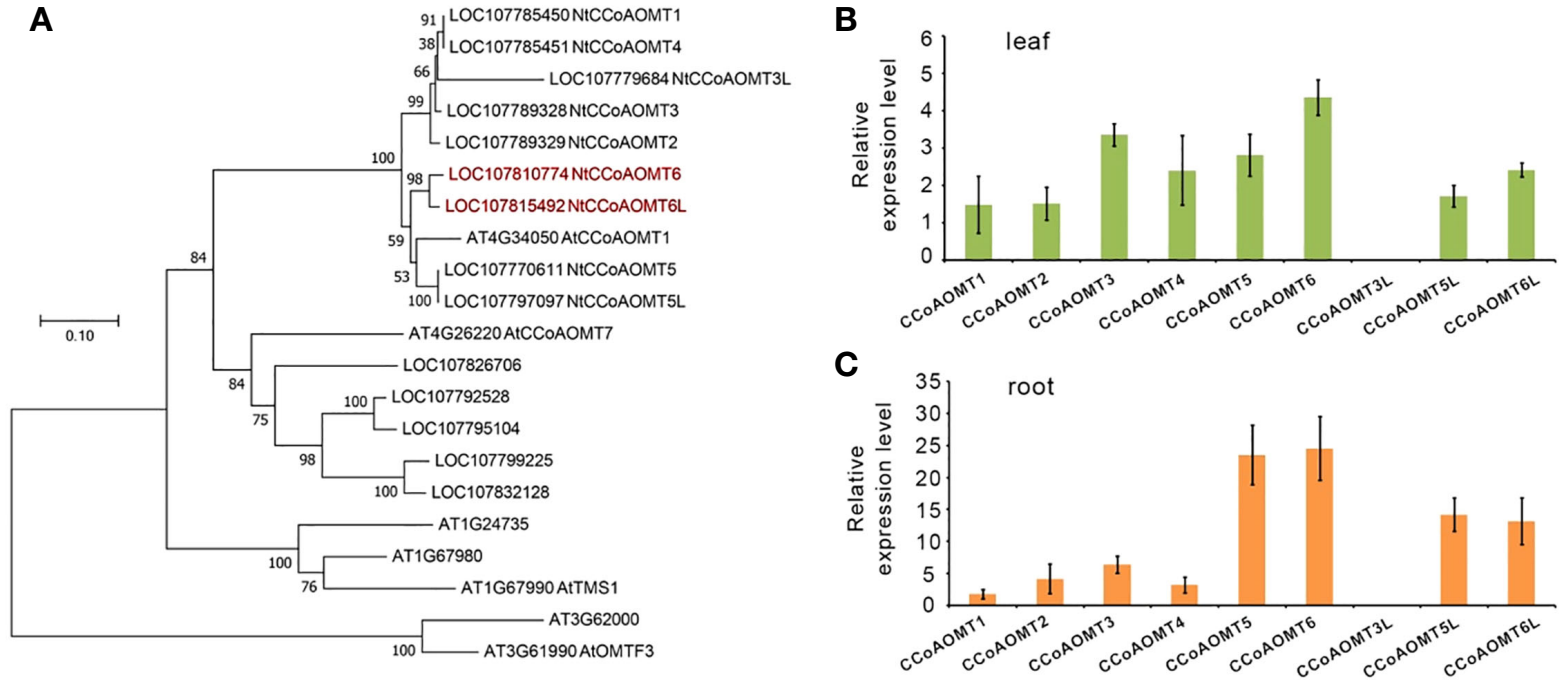
The CCoAOMT gene family in *Nicotiana tabacum.* **(A)** The phylogenetic tree of the CCoAOMT gene family in *Nicotiana tabacum* and *Arabidopsis thaliana*. A neighbor-joining (NJ) tree is reconstructed using the MEGA program version 7.0. Sequences are aligned by Muscle. Bootstrap values (expressed as percentages of 1,000 replications) are shown at branch points. **(B)** Relative expression analysis of the primary *CCoAOMTs* in the leaf. **(C)** Relative expression analysis of the primary *CCoAOMTs* in the root.

There were 14 predicted *NtCCoAOMT* genes that were searched from the *Nicotiana tabacum* genome in the NCBI database, of which nine genes were explicitly annotated (**Figure 1A**). The NtCCoAOMT family generally contains around 240 amino acids, all of which contain the caffeoyl-CoA O-methyltransferase domain (PLN02589 or PLN02781). A phylogenetic tree was constructed using seven AtCCoAOMTs from *Arabidopsis thaliana* and the above 14 *NtCCoAOMTs*. The results showed that LOC107826706, LOC107792528, LOC107795104, LOC107799225, and LOC107832128 were clustered together with *AtCCoAOMT7* (At4g26220), whereas *NtCCoAOMT1-6, 3L, 5L, 6L* clade with *AtCCoAOMT1* (At4g34050), which was confirmed to be the key member involved in the monolignol pathway (Do et al., 2007) (**Figure 1A**). Furthermore, we analyze the tissue expression level of these nine *NtCCoAOMTs* by qRT-PCR and found that *NtCCoAOMT6* was the most highly expressed in tobacco roots and leaves (**Figures 1B, C**), suggesting its critical position in the monolignol biosynthesis. *NtCCoAOMT6L* shares 95.7% sequence identity and similar expression patterns with *NtCCoAOMT6*. Because the common tobacco is an allotetraploid formed by combining different chromosomes of two wild species *N. sylvestris* and *N. tomentosiformis* (Sierro et al., 2014), we compared *NtCCoAOMT6* and *NtCCoAOMT6L* in these two wild species, respectively. It shows that *NtCCoAOMT6* originates from *N. tomentosiformis* (99% identities), whereas *NtCCoAOMT6L* originates from *N. sylvestris* (100% identities), which means they are orthologous genes from different subgenomes. Thus, we created loss-of-function mutants for these two genes in *Nicotiana tabacum*.

### Generation and characterization of double mutant *ccoaomt6 ccoaomt6l*

**Figure 2:**
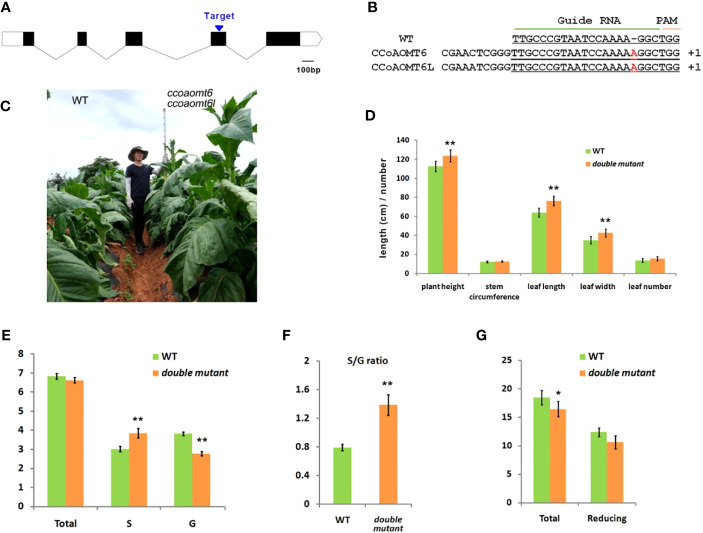
Sequence and phenotype of the CRISPR/Cas9 mutant. **(A)** Diagram of *CCoAOMT6* and *CCoAOMT6L* showing the target site for CRISPR/Cas9 technology. The CDS and UTR regions are indicated by the black box and white box, separately. **(B)** The sequence in the *ccoaomt6ccoaomt6l* double mutant. **(C)** The appearance of the WT (left) and *ccoaomt6ccoaomt6l* double mutant (right). **(D)** The agronomic traits of WT and *ccoaomt6ccoaomt6l* double mutant. The data are presented as mean ± s.d., n = 10. **(E)** Determination of total lignin and lignin monomer content. **(F)** Determination of the S/G ratio between the wild-type and double mutant. **(G)** Determination of total sugar and reducing sugar content. The data are presented as mean ± s.d., n = 5. *P < 0.05, **P < 0.01, Student’s tests.

We used CRISPR/Cas9 technology to generate a *ccoaomt6 ccoaomt6l* double mutant. A common sgRNA target (5′-TTGCCCGTAATCCAAAAGGCTGG-3′) in the two genes was selected (**Figure 2A**). The CRISPR construct was transformed into wild-type *Nicotiana tabacum*, and the homozygous double mutant was obtained. The double mutant contained a nucleotide insertion in the target sites of *CCoAOMT6* and *CCoAOMT6L*, leading to frameshift mutation, separately (**Figure 2B**). Under natural conditions, compared with the wild type, the mutant has a longer squaring stage, darker green leaves, and vigorous growth and development (**Figure 2C**). Statistical analysis showed that the plant height, leaf length, and leaf width were significantly increased (p < 0.01) (**Figure 2D**).

### Knockout of *CCoAOMT6* and *CCoAOMT6L* increases the S/G ratio of lignin monomers

CCoAOMT is the key methylation enzyme of caffeoyl-CoA, which produces feruloyl-CoA, and is the upstream component of G-lignin and S-lignin monomers. Therefore, we also detected the total lignin content and lignin monomer content in the wild type and the double mutant. The results showed that there was no significant difference in total lignin content between the mutant and the control; however, S-lignin content increased by around 27.5% and G-lignin content decreased by around 24.3% (**Figure 2E**), thus increasing the S/G ratio by nearly 68.4% (**Figure 2F**). The above results prove that tobacco CCoAOMT can control the type of lignin monomer by influencing the step of caffeoyl-CoA converting to feruloyl-CoA.

Furthermore, we detected the content of total sugar and reducing sugar that are important for plant growth and development. Compared with the wild type, the total sugar content in the double mutant leaves decreased by 11.0%, and the reducing sugar content decreased by 14.3% (**Figure 2G**), indicating that the decomposition of sugar may have promoted the synthesis of lignin monomers. In general, the mutation of *CCoAOMT6* and *CCoAOMT6L* has improved the economic traits of tobacco.

### Knockout of *CCoAOMT6* and *CCoAOMT6L* improves tobacco disease resistance

**Figure 3:**
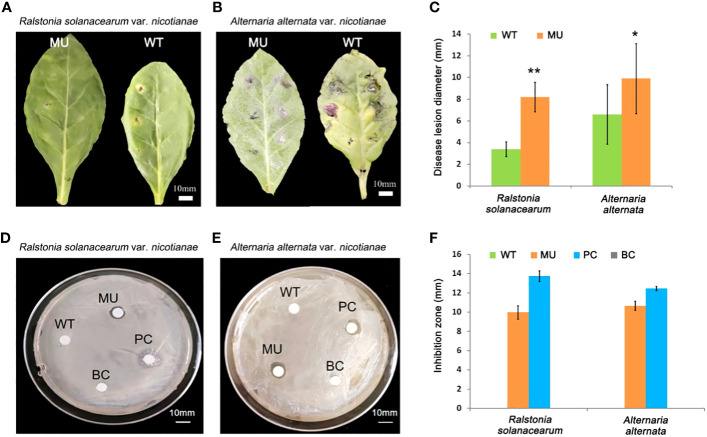
Disease resistance test of the CRISPR/Cas9 mutant. **(A, B)** Pathogenicity of tobacco bacterial wilt disease **(A)** and brown spot disease **(B)**. Bars = 10 mm. **(C)** Statistics of the disease lesion diameter against tobacco bacterial wilt disease and brown spot disease. MU, the double mutant; WT, the wild type. **(D, E)** Inhibition test of *Ralstonia solanacearum* **(D)** and *Alternaria alternata* **(E)**. MU, double-mutant tobacco leaf extract; WT, WT tobacco leaf extract; PC, 1 mg/mL cefotaxime sodium–95% ethanol solution (positive control); BC, 95% ethanol (blank control). **(F)** Statistics of the inhibition zone diameter against tobacco bacterial wilt disease and brown spot disease. Values show as mean ± s.d., n = 5. *P < 0.05, **P < 0.01, Student’s tests.

Bacterial wilt and brown spot are the main diseases of tobacco plants, caused by *Ralstonia solanacearum* infection and *Alternaria alternata* infection, respectively. To further study the effect of the changes in lignin monomer composition caused by *CCoAOMT6* and *CCoAOMT6L* knockout on tobacco disease resistance, *Ralstonia solanacearum* and *Alternaria alternata* strains were inoculated on the wild-type and double-mutant leaves, respectively, according to the methods of Guo et al. (2004)and Ma et al. (2017). The disease lesion diameter around the inoculation sites was measured after 72 h of inoculation. It was found that on the wild-type tobacco leaves, the bacterial wilt disease symptoms were obvious and the brown spot disease symptoms developed rapidly. For the mutant leaves, however, the bacterial wilt disease either did not appear or developed slowly, and the development of brown spot diseases disease symptoms is relatively slow (**Figures 3A–C**).

Similarly, we also investigated the inhibitory effect of transgenic tobacco leaf extract on the growth of *Ralstonia solanacearum* and *Alternaria alternata*, according to the protocol provided by Guo et al (Guo et al., 2004). The results showed that the ethanol extract of transgenic tobacco leaves indeed inhibited the growth of *Ralstonia solanacearum* and *Alternaria alternata*, whereas the extract of wild-type leaves did not (**Figures 3D–F**). This indicates that compounds in the mutant tobacco extract has a “chemical defense” response to *Ralstonia solanacearum* and *Alternaria alternata* pathogens. Taken together, it is proved that the knockout of *CCoAOMT6* and *CCoAOMT6L* can enhance the disease resistance of tobacco.

### Knockout of *CCoAOMT6* and *CCoAOMT6L* altered the primary and secondary metabolites

To investigate the effects of S/G-lignin ratio changes on the whole plant metabolism, widely targeted metabolome sequencing was performed between the leaves and roots of the wild type and double mutants. A total of 266 metabolites were detected, including carbohydrates, amino acids and derivatives, organic acids and derivatives, lipids, terpenes and sterols, phenolic acids, nucleosides, flavonoid, and coumarin; different metabolites of roots and leaves were screened (**Tables S1**, **S2**).

**Figure 4:**
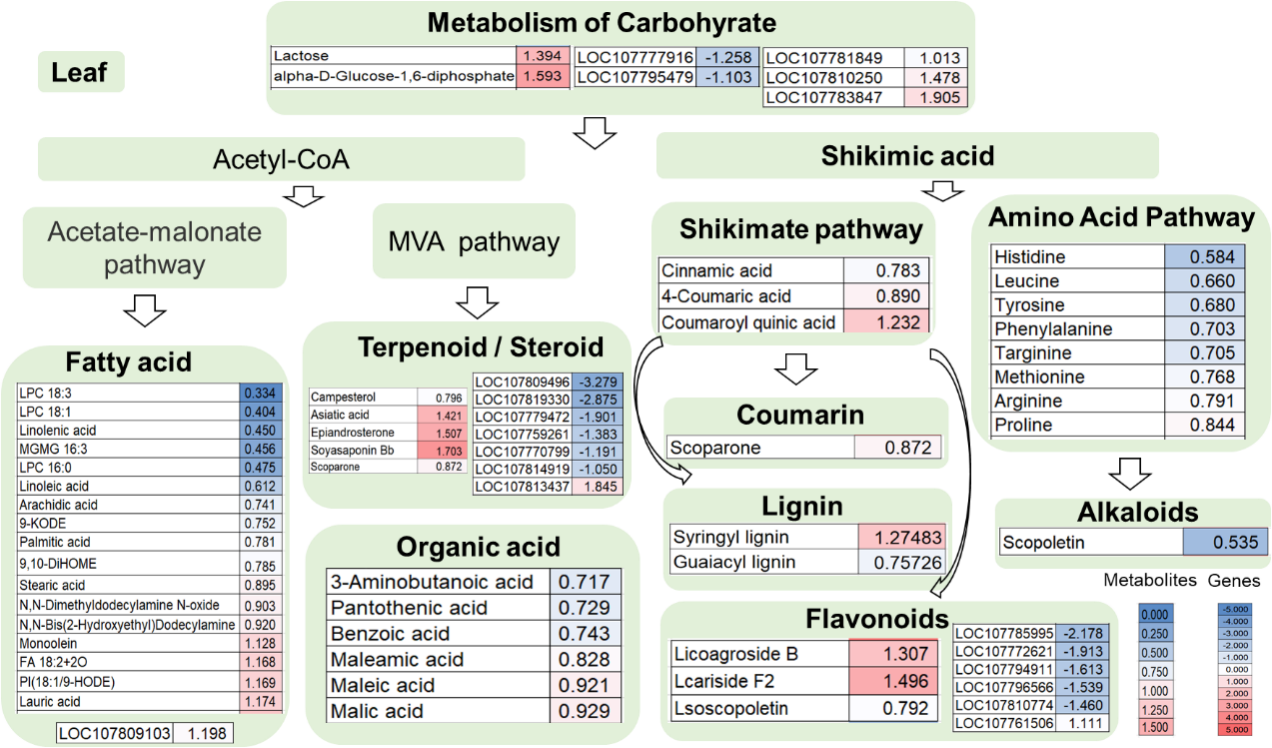
Leaf transcriptome combined with metabolome analysis in the double mutant. The heat map scale represents the relative level of metabolites and gene expression.

Compared with the wild type, 56 metabolites were differently detected from leaves of the double mutant, i.e., 15 upregulated and 41 downregulated (**Figure S2A**). Among them, eight differential amino acid and derivatives (eight downregulated) were identified, namely, histidine, leucine, tyrosine, phenylalanine, targinine, methionine, arginine, and proline. Moreover, there were 17 differential metabolites in the fatty acid pathway, of which four were upregulated and 13 were downregulated. Also, six organic acids were identified and all of them downregulated in the mutant line. There were five differential terpenoids or steroids identified, including two downregulated (campesterol, scoparone) and upregulated (asiatic acid, epiandrosterone, soyasaponin Bb). It also included nine metabolites involved in the shikimate pathway (five downregulated and four upregulated), namely, cinnamic acid, 4-coumaric acid, coumaroylquinic acid, one coumarin, two lignin, and three flavonoids. Scopoletin belongs to coumarin, which was downregulated in the mutant. Lactose and alpha-D-glucose-1,6-diphosphate were involved in the carbohydrate metabolic pathway, both of which were upregulated in the mutant (**Figure 4**).

**Figure 5:**
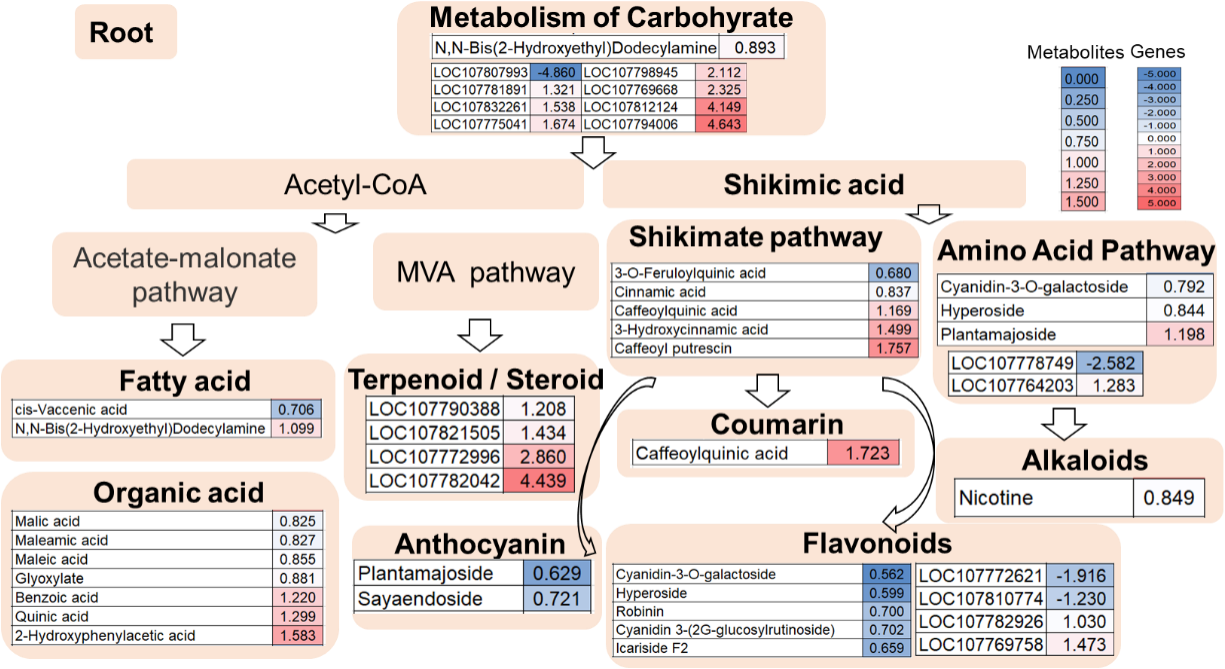
Root transcriptome combined with metabolome analysis in the double mutant. The heat map scale represents the relative level of metabolites and gene expression.

Meanwhile, 32 differential metabolites were identified from the roots between the double mutant and the wild type, of which 11 were upregulated and 21 were downregulated (**Figure S2B**). Among them, three differential flavonoid and derivatives (two down and one up) were identified, namely cyanidin-3-O-galactoside, hyperoside, and plantamajoside. Also, two metabolites were found in the fatty acid pathway, of which one was upregulated and one was down-regulated. Moreover, seven organic acids were identified of which three were upregulated and four were downregulated and 13 differential metabolites in the shikimate pathway (nine down and four up) were identified, one coumarin (caffeoylquinic acid up), two anthocyanin (two down), and five flavonoids (two down and three up). Nicotine was also identified, which was downregulated in the mutant line. N,N-Bis (2-hydroxyethyl)dodecylamine was involved in carbohydrate metabolism, which was downregulated in the mutant line (**Figure 5**).

### Knockout of *CCoAOMT6* and *CCoAOMT6L* altered the gene expression in the primary and secondary metabolic pathways

To better analyze the potential roles of *CCoAOMT6* and *CCoAOMT6L* in primary and secondary metabolic regulation, we conducted transcriptome profiling of the leaves and roots in the double mutant and the wild type. A total of 248 DEGs (differentially expressed genes) were detected in the leaf data, which were composed of 226 genes that were upregulated and 22 genes that were downregulated (**Figures S3A, B**). Moreover, 332 DEGs were detected in the root data, composed of 149 upregulated genes and 183 downregulated genes (**Figures S3A, C**). Only 38 DEGs were identified in both leaves and root, suggesting that the functions of *CCoAOMT6* and *CCoAOMT6L* in different tissues were slightly different (**Figure S3A**).

All the DEGs were annotated according to GO, KEGG, TF, COG, uniProt, InterPro, and WoLF PSORT. The GO terms “protein folding,” “protein complex oligomerization,” “response to hydrogen peroxide,” and “response to reactive oxygen species” are the most highly enriched in both leave and root data (**Figure S4**). KEGG analysis of the leaf data showed that “protein processing in endoplasmic reticulum,” “vitamin B6 metabolism,” “stilbenoid, diarylheptanoid and gingerol biosynthesis,” “sesquiterpenoid and triterpenoid biosynthesis,” and “flavonoid biosynthesis” were significantly enriched (**Figure S5A**). Meanwhile, KEGG analysis of the root data showed that “protein processing in endoplasmic reticulum,” “galactose metabolism,” “diterpenoid biosynthesis,” “ascorbate and aldarate metabolism,” and “stilbenoid, diarylheptanoid and gingerol biosynthesis” were significantly enriched (**Figure S5B**).

In the leaf data shown in **Figure 4**, among primary metabolite pathway biosynthesis enzyme genes, the expression of transcripts coding for carbohydrate such as galactinol synthase 2-like (LOC107783847), galactinol-sucrose galactosyltransferase-like (LOC107810250), and 1,4-alpha-glucan-branching enzyme 3 (LOC107781849) were upregulated and mannan endo-1,4-beta-mannosidase 7-like (LOC107777916) and probable isoaspartyl peptidase/L-asparaginase 3 (LOC107795479) were reduced. Fatty acid elongation gene LOC107809103 in the fatty acid pathway was upregulated (**Figure 4**). Among flavonoid biosynthesis enzyme genes, the transcripts coding for feruloyl CoA ortho-hydroxylase 1-like (LOC107796566), caffeoyl-CoA O-methyltransferase 6-like (LOC107772621), agmatine coumaroyltransferase-2-like (LOC107794911), caffeoyl-CoA O-methyltransferase 6 (LOC107810774), and trans-resveratrol-di-O-methyltransferase-like (LOC107785995) were downregulated whereas the transcript coding for agmatine coumaroyltransferase-2-like (LOC107761506) was upregulated (**Figure 4**). In addition, seven genes involving the terpenoid/steroid biosynthesis enzyme gene were detected by transcriptome profiling. Dehydrodolichyl diphosphate synthase 6-like (LOC107814919), 3-hydroxy-3-methylglutaryl-coenzyme A reductase-like (LOC107770799), ent-copalyl diphosphate synthase, chloroplastic-like (LOC107809496), 5-epiaristolochene 1,3-dihydroxylase (LOC107759261), alpha-farnesene synthase-like (LOC107779472), and probable terpene synthase 3 (LOC107819330) were downregulated, while the transcript coding for gibberellin 2-beta-dioxygenase 1-like (LOC107813437) was upregulated (**Figure 4**).

In the root data shown in **Figure 5**, among primary metabolite pathway biosynthesis enzyme genes, the expressions of transcripts coding for carbohydrate such as galactinol synthase 1-like (LOC107812124), bifunctional UDP-glucose 4-epimerase and UDP-xylose 4-epimerase 1-like (LOC107775041), bifunctional UDP-glucose 4-epimerase and UDP-xylose 4-epimerase 1-like (LOC107781891), acid beta-fructofuranosidase-like (LOC107807993), beta-amylase 1, chloroplastic-like (LOC107832261), alpha-amylase-like (LOC107769668), and alpha-galactosidase 3-like (LOC107798945) were upregulated and acid beta-fructofuranosidase-like (LOC107807993) was reduced. 1-(5-Phosphoribosyl)-5-[(5-phosphoribosylamino)methylideneamino] imidazole-4-carboxamide isomerase (LOC107778749) and homogentisate 1,2-dioxygenase-like (LOC107764203) in the amino acid pathway were down- and upregulated, respectively (**Figure 5**). Among flavonoid biosynthesis enzyme genes, the transcripts coding for caffeoyl-CoA O-methyltransferase 6 (LOC107810774) and caffeoyl-CoA O-methyltransferase 6-like (LOC107772621) were downregulated whereas the transcript coding for a4-coumarate-CoA ligase 2-like (LOC107769758) and peroxidase P7-like (LOC107782926) were upregulated (**Figure 5**). In addition, four genes involving terpenoid/steroid biosynthesis enzyme genes were detected by transcriptome profiling. The expression of transcripts coding for 8-hydroxygeraniol dehydrogenase-like (LOC107772996), gibberellin 2-beta-dioxygenase 1-like (LOC107782042), gibberellin 2-beta-dioxygenase 1-like (LOC107821505), and gibberellin 2-beta-dioxygenase 1-like (LOC107790388) were upregulated (**Figure 5**).

### Knockout of *CCoAOMT6* and *CCoAOMT6L* upregulated metabolite docking study for possible chemical mechanism of tobacco disease resistance

**Figure 6:**
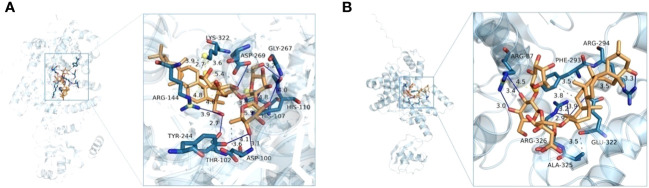
Soyasaponin Bb in the tyrosyl-tRNA synthetase active sites of *Ralstonia solanacearum* **(A)** and *Alternaria alternata* **(B)**.

The above bacteriostatic experiments showed that there were some antimicrobial agents in transgenic tobacco leaves. Because phenolic acids, coumarins, terpenoids, and other compounds have good biological activities, we screened six compounds such as icariside F2, epiandrosterone, soyasaponin Bb, 3-hydroxycinnamic acid, esculin, and 4-coumaroylcholine from upregulated metabolites (the threshold is 1.5-fold). Aminoacyl-tRNA synthetases (aaRSs) play a key role in protein biosynthesis, and the inhibition of aaRSs affects cell growth (Pisano et al., 2019). The tyrosyl-tRNA synthetase 3D structures of *Ralstonia solanacearum* and *Alternaria alternata* were obtained from UniProt (https://www.uniprot.org/). Mupirocin, which target to tyrosyl-tRNA synthetase, is a good inhibitor of pathogens as a reference molecule (Francklyn and Mullen, 2019). Molecular docking results are shown in **Table 1** and **Figure 6**. From **Table 1**, it can be found that the binding energies of A with tyrosyl-tRNA synthetase of *Alternaria alternata* and *Ralstonia solanacearum* are −15.469 and −14.748 kcal/mol, respectively, which are better than those of reference molecules mupirocin’s −9.540 and −8.186 kcal/mol, respectively. As shown in **Figure 6**, soyasaponin Bb can interact with tyrosyl-tRNA synthetase of *Alternaria alternata* residues ASP-100, THR-102, HIS-110, ARG-144, TYR-244, ASP-269, GLY-267, and ARG-144 form nine hydrogen bonds. It forms three hydrophobic interactions with ASP-100 and LYS-322. Moreover, soyasaponin Bb can form six hydrogen bonds with residues ARG-87, ARG-294, and ARG-326 and form four hydrophobic interactions with residues PHE-293, ARG-294, and ALA-325 in tyrosyl-tRNA synthetase of *Ralstonia solanacearum* (**Table 1** and **Figure 6**). It indicates that soyasaponin Bb may have a good inhibitory effect on *Ralstonia solanacearum* and *Alternaria alternata* and upregulation of soyasaponin Bb may be a chemical reason for the improvement of disease resistance in the mutant tobacco. Of course, other upregulated metabolites have high binding energy with tyrosyl-tRNA synthetase (<−5 kcal/mol), which may also enhance the tobacco resistance (**Table 1**). It may also be that the synergism of upregulated metabolites improves the disease resistance of tobacco.

**Table 1:** The tyrosyl-tRNA synthetases are docked with upregulated metabolites.

Compounds	*Alternaria alternata*			*Ralstonia solanacearum*		
	Hydrogen bond(s)	Binding energy (kcal/mol)	Residue involved H-bond	Hydrogen bond(s)	Binding energy (kcal/mol)	Residue involved H-bond
**Soyasaponin Bb^L^**	9	−15.469	ASP-100; THR-102; HIS-110; ARG-144; TYR-244; ASP-269; GLY-267 ARG-144(2)	6	−14.748	ARG-87(2); ARG-294; ARG-326(3)
**Icariside F2^L^**	5	−7.367	GLY-109; PHE-320; GLY-267; GLY-98; ASP-269	10	−8.117	GLN-201; ASP-218; ASP-109; TYR-197(3); GLN-219; SER-111; ASP-69(2)
**Epiandrosterone^L^**	4	−8.006	PHE-320(2); LEU-312; GLN-270	2	−8.167	LEU-246; GLY-67
**Esculin^R^**	8	−7.706	GLY-98; PRO-310; LEU-312(3); ASP-269; PHE-320; GLY-267	8	−8.153	TYR-197; GLN-219; GLY-216; GLY-78(2); MET-255(2); LEU-246
**4-Coumaroylcholine^R^**	1	−4.546	PHE-96	2	−6.122	GLY-78; LEU-246
**3-Hydroxycinnamic acid^R^**	2	−6.016	GLN-52; ARG-92	4	−5.644	GLY-112; ASP-178(2); PHE-221
**Mupirocin (reference)**	1	−9.540	TYR-176	2	−8.186	TRY-176; ARG-177

^L^The upregulated metabolites of tobacco leaf.

^R^The upregulated metabolites of tobacco root.

## Discussion

In plants, COMT and CCoAOMT are methylation enzymes at two different substrate levels in the lignin biosynthetic pathway, giving rise to two parallel pathways. Compared with COMT, the role of CCoAOMT in the lignin biosynthesis pathway was studied relatively late. At present, *CCoAOMT* has been successfully cloned from pomelo, poplar, and other plants and the overexpression plants have been constructed (Liu et al., 2014; Xu et al., 2014). Some studies have found that *COMT* and *CCoAOMT* have relatively consistent high expression in cotton root, stem, and other vascular tissues (Lv et al., 2010). In this study, 14 CCoAOMT genes were identified in tobacco, of which *CCoAOMT5/5L* and *CCoAOMT6/6L* were highly expressed in leaves and roots, especially in roots, suggesting that they may play a key role in tobacco lignin biosynthesis (**Figure 1**).

In recent years, more and more studies have been conducted on the regulation of lignin content in plants by inhibition of CCoAOMT activity. Inhibiting the expression of *CCoAOMT* will inhibit the biosynthesis of S-monomer lignin or G-monomer lignin to a varying extent, especially G-monomer, so as to control the S/G ratio. It is found that the lignin content in transgenic poplars with inhibited *CCoAOMT* expression decreased by 12% and the S/G ratio increased by 11% (Meyermans et al., 2000). Zhong et al. transferred the antisense-CCoAOMT into poplar and found that the lignin content decreased significantly, and the loose structure was conducive to delignification (Zhong et al., 2000). The difference is that this study found that the knockout of *CCoAOMT6/6L* in tobacco does not affect the content of total lignin but increases and decreases the content of S-monomer and G-monomer to increase the S/G ratio by 68.4%, and the plant growth and development are better (**Figures 2**, **3**). On the one hand, this may be due to its functional redundancy with *CCoAOMT5/5L* to ensure plant growth; on the other hand, it shows the importance of *CCoAOMT6/6L* in regulating the S/G ratio. However, the reduction of total sugar content may ensure the metabolic balance of this lignin fine-tuning (**Figure 3**).

The combined analysis of transcriptome and metabolism showed that the functions of *CCoAOMT6/6L* in roots and leaves were similar, both of which mainly affected the primary and secondary metabolism. However, the upregulation of primary metabolism and terpenoid metabolism genes in roots is more obvious whereas the phenylpropane metabolism pathway is mainly affected in leaves (**Figures 4**, **5**, **S5**). This result is consistent with the phenotype. The increase of primary metabolism in roots may promote plant growth due to the sink–source relationship (Caretto et al., 2015). The phenylpropane metabolic pathway also plays an important role in plant–microorganism interaction (Naoumkina et al., 2010). Lignin biosynthesis of *Verticillium dahliae*-resistant tomato line LA3038 increased (Gayoso et al., 2010). After cotton hypocotyls were treated with *V. dahliae*, the biosynthesis of lignin and lignin phenolic polymers increased, and the expression of phenylpropanoid pathway genes, such as chalcone synthase (CHS) and phenylalanine ammonia-lyase (PAL), was upregulated (Smit and Dubery, 1997). In addition, terpenoid metabolic pathways are also closely related to plant growth, development, and resistance. The triterpene metabolism pathway in *Arabidopsis thaliana* can specifically regulate the composition of rhizosphere microorganisms to affect plant growth and development (Huang et al., 2019). Triterpenoids in rice tapetum can regulate the formation of pollen coat (Xue et al., 2018). The triterpene saponins in oat root have antibacterial function, which can prevent soil borne diseases, such as take-all disease (Li et al., 2021).

On the other hand, lignin plays an important role in the plant defense response to variant diseases. In previous reports, S lignin inhibits the infection and reproduction of nematodes in *Arabidopsis* and tobacco (Wuyts et al., 2006) and S lignin will accumulate in the process of wheat-hypersensitive resistance (Menden et al., 2007). In Ma’s work, total lignin and S lignin were considered to be the main factors contributing to the basic defense response of tobacco (Ma et al., 2017). Ma believes that S lignin is only developed in angiosperms and S lignin is evolved by plants to cope with more complex disease-resistant environments. Long et al. studied the defense effect of tobacco against *Alternaria alternata* and *Phytophthora nicotianae* pathogens and showed that the induced expression of phenylalanine biosynthesis and sesquiterpenoid biosynthesis pathway synthesis genes, resulting in phytoalexin scopoletin and capsidiol, played a chemical defense role (Long et al., 2021). A triterpenoid saponin, soyasaponin has anticancer, antioxidant, and antiviral activities and has a different influence on the oil bacterial genera (Sugiyama, 2019; Fujimatsu et al., 2020; Nakayasu et al., 2021). According to the results of molecular docking (**Table 1** and **Figure 6** and ), the upregulated compounds such as soybean soyasaponin Bb, as chemical defense, enhance the defense response to tobacco varieties. Considering the previous reports, in addition to S lignin, upregulated metabolisms like soyasaponin Bb are other important factors that contribute to the basic defense response in tobacco. Therefore, it is speculated that the growth phenotype of the double mutants may be caused by the perturbation of phenylpropanoid and terpenoid metabolisms by the fine-tuning of lignin monomers. In addition, S lignin features an extra methoxy functional group compared with G lignin, thereby leading to a heightened concentration of active moieties within the double mutant. This particular attribute holds the potential to play a role in enhancing the growth and developmental outcomes of the double mutant line (Parra et al., 2011).

## Conclusions

In total, we have provided the physiological and molecular evidence to demonstrate that CCoAOMT6 and CCoAOMT6L are involved in growth development and disease resistance by adjusting the S/G ratio of lignin monomers in *Nicotiana tabacum*. Furthermore, the combined analysis of metabolome and transcriptome proved that these phenotypes may be caused by affecting phenylpropane and terpene metabolism in the leaves and roots. In addition to S lignin, upregulated compounds such as soyasaponin Bb may enhance the tobacco disease defense. Our research provides a theoretical basis for the improvement of tobacco varieties.

## Methods

### Identification and phylogenetic analysis

To identify potential members of the *CCoAOMT* gene family in *Nicotiana tabacum*, we performed NCBI database searches. Seven *Arabidopsis CCoAOMT* sequences were used as queries in BLAST searches against the *Nicotiana tabacum* genome sequence (https://www.ncbi.nlm.nih.gov/genome/?term=Nicotiana+tabacum). The exons and introns were analyzed and calculated by IMEter v2.0 (Wu et al., 2023). SMART toolkit (http://smart.embl-heidelberg.de/) was used for protein domain analysis.

Full-length protein sequences of 14 tobacco *CCoAOMTs* and 7 *Arabidopsis CCoAOMTs* were used for building the phylogenetic tree. Multiple-sequence alignments were performed using Muscle 3.52. A neighbor-joining (NJ) tree is reconstructed using the MEGA program version 7.0. Support for each node was tested with 1,000 bootstrap replicates.

### RNA extraction and cDNA synthesis

The total RNA of the wild type and the double mutant was isolated respectively from finely ground tissue samples of leaves and roots of tobacco growing in the open field in Kunming (2021), China. We used the BGI-NGS-TQ-RNA-005 A0 Kit (BGI) for the extractions. The purity and integrity of the RNA were evaluated through gel electrophoresis and the ratios of A_260_/A_230_ and A_260_/A_280_. The qualified RNA is used for subsequent reverse transcription and transcriptome library construction. We used the TransScript II First-Strand cDNA Synthesis Kit (TransGen) for cDNA reverse transcription.

### Real-time PCR

To analyze gene expression levels, a qRT-PCR assay was completed with SYBR Green (Takara) and a 7500 Real-Time PCR system (Life Technologies).The reaction mixture was 20 μL, including SYBR Premix Ex Taq 10 μL, 10 μM reverse-specific primer 0.4 μL, and 0.1 μM cDNA templates 2 μL. Actin was used as a reference gene. The 2^−△△CT^ method was used to analyze the relative gene expression (Livak and Schmittgen, 2001). The experiment was repeated three times, and the primers are listed in **Table S3**.

### Plasmid construction

The binary vector pORE-Cas9 was used as backbone like that previously described (Gao et al., 2015; Gao et al., 2018), which contains the CRISPR/Cas9 components. The 25-bp oligos (F: GATTGTTGCCCGTAATCCAAAAGGC; R: AAACGCCTTTTGGATTACGGGCAAC) with GAAT and AAAC overhands, respectively, were annealed and then inserted into the BsaI site of the pORE-Cas9 binary vector by T7 ligase.

### Creation of the double mutant using CRISPR/Cas9 technology

The pORE-Cas9 binary vectors containing the specific guide RNA and Cas9 expression cassettes were transformed into *Agrobacterium tumefaciens* LBA4404 by the freeze–thaw method. The positive clones were then used to produce target gene mutant tobacco plants with the leaf discs method (Horsch et al., 1985). Kanamycin-resistant seedlings were obtained, and mutants were identified.

### Lignin content analysis

The total lignin contents of dried tobacco leave and root tissues were quantitatively determined by using the Klason method (Eudes et al., 2006; Ma et al., 2017). Lignin monomer composition was measured using thioacidolysis (Zhao et al., 2010; Ma et al., 2017).

### Total sugar and reducing sugar analysis

Determination methods of total sugar and reducing sugar followed protocols of Tobacco Trade Standards (China National Tobacco Quality Supervision&TestCenter, 2002).

### Transcriptome sequencing

Extracted RNA was assessed for quality and quantity using an Agilent 2100 Bioanalyzer (Agilent Technologies), and mRNA library preparation refers to the construction process BGI-NGS-JK-RNA-001 (https://www.yuque.com/yangyulan-ayaeq/oupzan/gu8ls7). The amplified libraries were sequenced on a DNBSEQ sequencing machine at BGI company according to Qi et al (Qin et al., 2020). The sequencing data were filtered with SOAPnuke (Li et al., 2008). Bowtie2 (Langmead and Salzberg, 2012) was applied to align the clean reads to the gene set. The gene expression level was calculated by RSEM (v1.3.1) (Li and Dewey, 2011). Differential expression analysis was performed using the DESeq2 (v1.4.5) (Love et al., 2014) with log_2_FC ≥1 and Q value ≤0.05. All DEGs were annotated according to GO, KEGG, KOG, Swiss-Prot, and TrEMBL databases. There are six replicates in the leaf and root groups between WT and the mutant.

### Tobacco disease resistance test

Resistance against pathogens test. The leaves were the fourth leaf from the top, taken from the 2-month planted WT tobacco and double-mutant tobacco. The wound sites were scratched by the toothpick back of the leaf, evenly distributed on both sides of the main leaf vein. The challenged leaves were put into a 150-mm culture dish with water-soaked filter paper at the bottom and incubated at 28°C for 72 h, then the photoperiod was set as 16-h light and 8-h darkness.

Tobacco leaf extract inhibition test. 100 mg of MU and WT tobacco leaf-lyophilized powder was ultrasonically extracted for 40 min by 10 mL of 95% ethanol and filtered, and MU and WT extracts were obtained after filtration. The 6-mm-diameter filter paper was put into the MU extract, WT extract, 95% ethanol, and 1 mg/mL cefotaxime sodium ethanol solutions and soaked for 10 min. 100 μL of *Alternaria alternata* and *Ralstonia solanacearum* bacterial fluids was taken and coated in PDA and LB plate culture medium, respectively, then kept in a 28°C incubator for 24 h.

### Widely targeted metabolomic analysis

20.0 mg (accurate to 0.1 mg) sample powder was weighed, and 4 mL of methanol–water (v:v = 8:2) solution was added, shaken on a vortex mixer at 2,000 r/min for 30 s, sonicated in an ice-water bath for 30 min, centrifuged at 13,000 r/min for 15 min at 4°C, and passed through a 0.22-μm organic filter membrane, ready for analysis.

High-performance liquid chromatography (LC-20A, Shimadzu) tandem time-of-flight mass spectrometry (AB SCIEX 5600+, Shimadzu), and XSelect HSS T3 (4.6 × 150 mm, 3.5 µm) column chromatography were performed for the metabolomic analysis. The positive ionization mode mobile phase was 0.1% formic acid/water (A) and acetonitrile (B), and the negative ionization mode mobile phase was 5 mM ammonium formate (A) and acetonitrile (B). The mobile phase gradient elution program was: 0.00 min–3.00 min, 10%B; 3.00 min–21.00 min, 10%–95%B; 21.00 min–28.00 min, 95%B; 28.00 min–28.10 min, 10%B; 28.10 min–34.00 min, 10%B. The QTOF/MS scanning mode is an information-dependent (IDA) full-scan mode, the mass scanning range is m/z 50–1,000, the source voltages of the positive and negative ionization modes are 5,500 V and 4,500 V, respectively, and the curtain gas, atomizer (gas1), and heating gas (gas2) flow pressures are 25 psi, 50 psi, and 50 psi, respectively. The collision energies (CE) were 30 V and −30 V, respectively. Qualitative analysis of metabolites was based on the local database (Jiangsu Academy of Agricultural Sciences) and the public database of metabolite information, including NIST, METLIN (https://metlin.scripps.edu), and HMDB (https://hmdb.ca/). Metabolites with significant differences were set with thresholds of variable importance in projection (VIP) ≥1 and fold change ≥1 or ≤0.5. Eight biological replicates of each sample were performed in each group.

### Molecular docking

In this study, AutoDock Vina (Vina, version 1.1.2) is running with a semi-flexible docking method, with docking accuracy up to 78%. PyMOL (version 4.3.0) software (https://pymol.org/) was used for separation of original ligand and protein structure, dehydration, and removal of organic matter. AutoDockTools (http://mgltools.scripps.edu/downloads) is used for hydrogenation, checking the charge, designating the atom type as AD4 type, calculating the Gasteiger, and constructing the docking grid box of the protein structure. In addition, the chemical composition (small-molecule ligand) should be determined as the root, and the reversible bond of the ligand should be selected in AutoDockTools.

## Data availability statement

The datasets presented in this study can be found in online repositories. The names of the repository/repositories and accession number(s) can be found in the article/**Supplementary Material**.

## Author contributions

ML performed the experiments and contributed to the original manuscript. HL analyzed the data. JZ contributed to the plotting and data analysis. CL and YL contributed to the analysis and interpretation of the data and drafting and revision of the manuscript. GY, TX, and HH contributed to the analysis and interpretation of data. YX and WK contributed to the analysis and interpretation of data. BH, XQ, and JW contributed to the design and conduct of the study, and the drafting and revision of the manuscript. All authors contributed to the article and approved the submitted version.
