# Supplementary material for: Knockout of CAFFEOYL-COA 3-O-METHYLTRANSFERASE 6/6L enhances the S/G ratio of lignin monomers and disease resistance in Nicotiana tabacum
Source: Front Plant Sci. 2023 Oct 5;14:1216702. doi: 10.3389/fpls.2023.1216702 (PMC10585270; doi:10.3389/fpls.2023.1216702)
Supplement: Supplementary file 4 [file DataSheet_1.docx]

Supplementary Material

**Knockout of CAFFEOYL-COA 3-O-METHYLTRANSFERASE 6/6L enhances the S/G ratio of lignin monomers and disease resistance in *Nicotiana tabacum***

Mingxin Liu^1,2†^, Huayin Liu^1,3†^, Jianduo Zhang^1†^, Cui Li^4,5^, Yinke Li^2^, Guangyu Yang^1^, Tong Xia^4,5^, Haitao Huang^1^, Yong Xu^1^, Weisong Kong^1^, Bingzhu Hou^4,*,†^, Xiaoquan Qi^4,*,†^,Jin Wang^1,*,†^

Corresponding author, E-mail: wangjin@iccas.ac.cn.

1 Research and Development of Center, China Tobacco Yunnan Industrial Co., Ltd., Kunming 650231, China; 2 School of Ethnic Medicine, Yunnan Minzu University, Kunming 650504, China；3 Technology Center, China Tobacco Yunnan Industrial Co.，Ltd., Kunming 650106，China；4 Key Laboratory of Plant Molecular Physiology, Institute of Botany, Chinese Academy of Sciences, Nan XinCun 20, Fragrant Hill, Beijing 100093, China. 5 University of Chinese Academy of Sciences, Yu Quan Road 19, Beijing 100049, China.

**List of Figures**

**Supplemental Figure 1.** The relative expression levels of *CCoAOMTs* in tobacco stem extracted from the TN90 RNAseq dataset, as presented by Sierra et al. in 2014. FPKM (Fragments Per Kilobase of transcript per Million mapped reads) was used to represent the relative expression levels.

**Supplemental Figure 2** Differential metabolites analysis of leaves (A) and roots (B) between the wild-type and the double mutant *ccoaomt6 ccoaomt6l.*

**Supplemental Figure 3** Transcriptome analysis of leaves and roots between the wild-type and the double mutant *ccoaomt6 ccoaomt6l.*(A) A Venn diagram presentation of differentially expressed gene in *ccoaomt6 ccoaomt6l* compared with WT. (B) Number of the up- and down- regulated DEGs in the leaf data. (C) Number of the up- and down- regulated DEGs in the root data.

**Supplemental Figure 4** GO enrichment analysis of DEGs in leaves (A) and roots (B) between the wild-type and the double mutant *ccoaomt6 ccoaomt6l.*

**Supplemental Figure 5** KEGG classification of DEGs in leaves (A) and roots (B) between the wild-type and the double mutant *ccoaomt6 ccoaomt6l.*


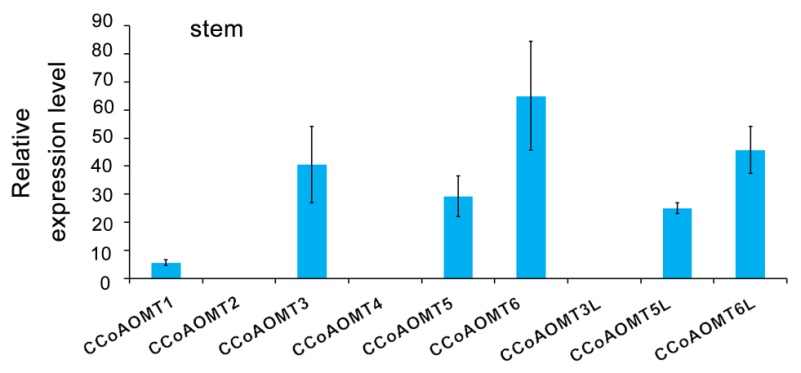


**Supplemental Figure 1.** The relative expression levels of *CCoAOMTs* in tobacco stem extracted from the TN90 RNAseq dataset, as presented by Sierra et al. in 2014. FPKM (Fragments Per Kilobase of transcript per Million mapped reads) was used to represent the relative expression levels.


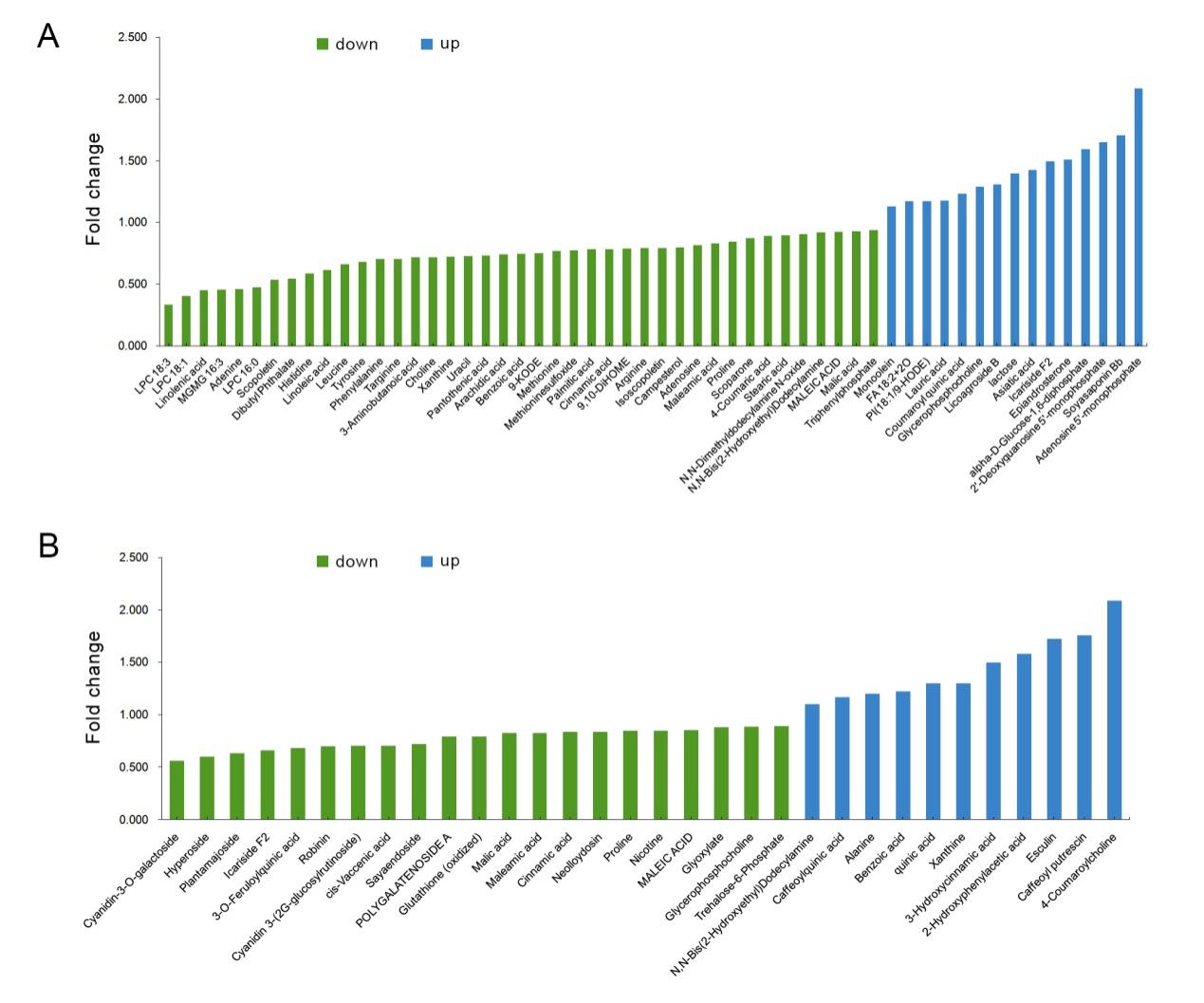


**Supplemental Figure 2.** Differential metabolites analysis of leaves (A) and roots (B) between the wild-type and the double mutant *ccoaomt6 ccoaomt6l*


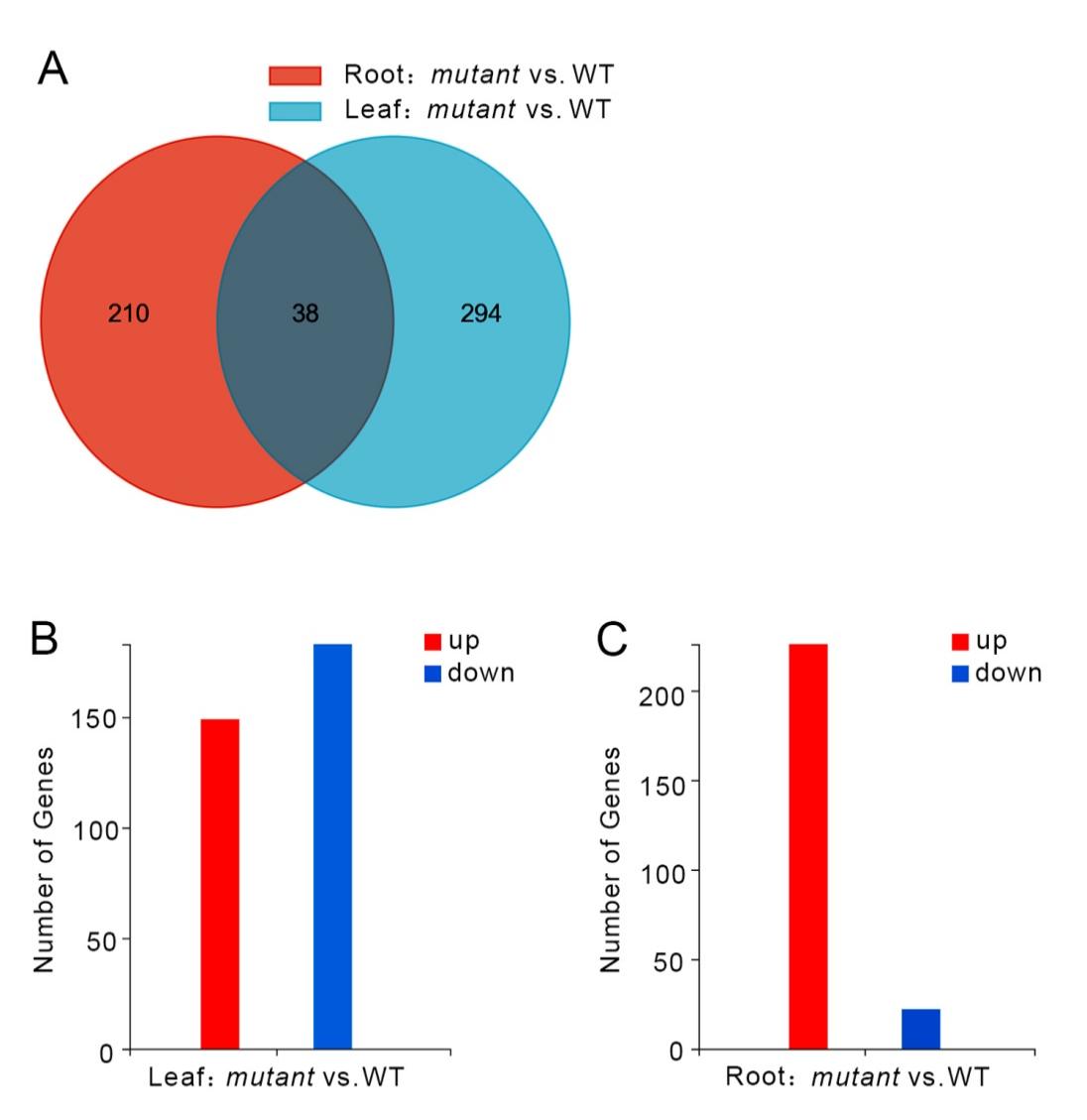


**Supplemental Figure 3.** Transcriptome analysis of leaves and roots between the wild-type and the double mutant *ccoaomt6 ccoaomt6l*

1. A Venn diagram presentation of differentially expressed gene in *ccoaomt6 ccoaomt6l* compared with WT. (B) Number of the up- and down- regulated DEGs in the leaf data. (C) Number of the up- and down- regulated DEGs in the root data.


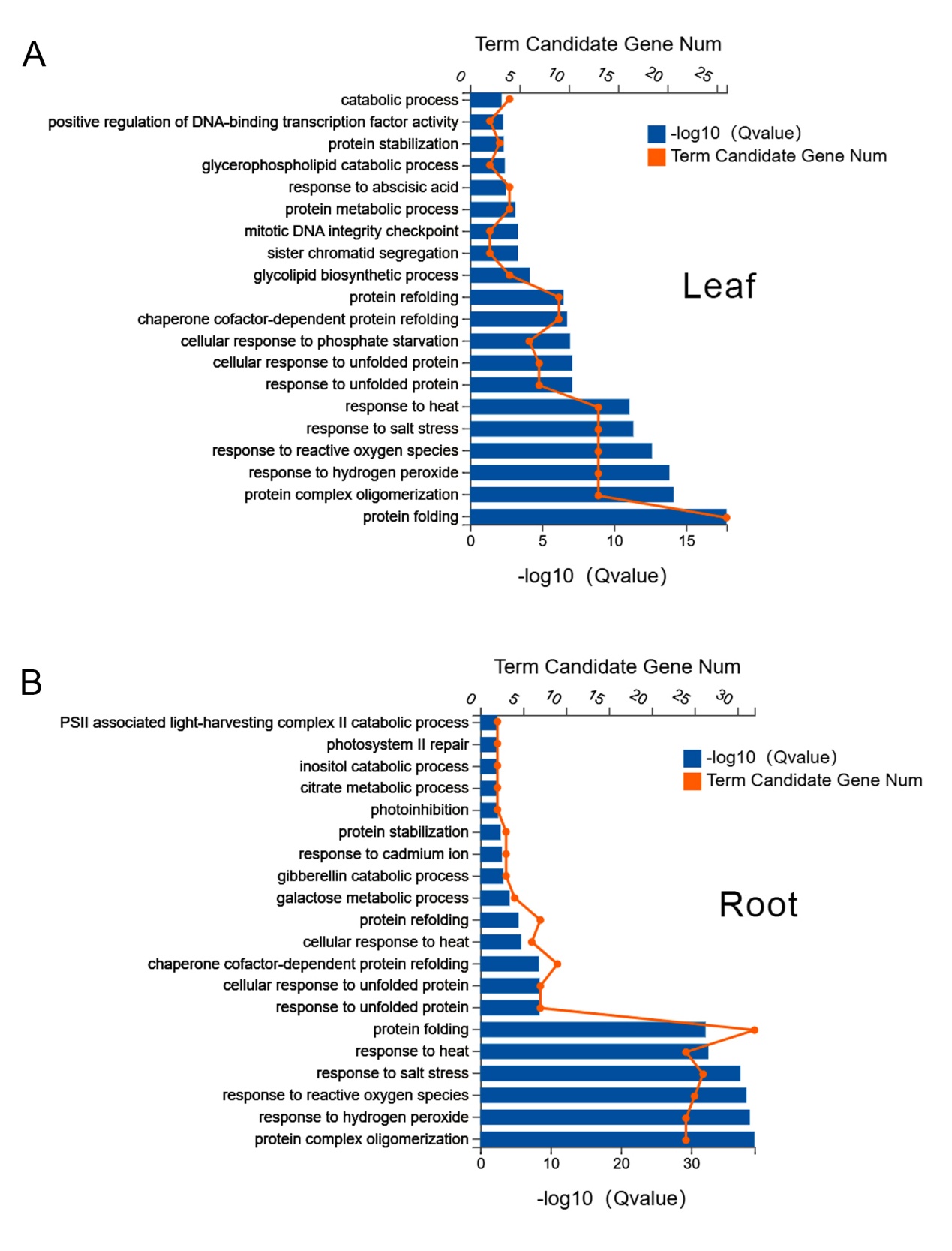


**Supplemental Figure 4.** GO enrichment analysis of DEGs in leaves (A) and roots (B) between the wild-type and the double mutant *ccoaomt6 ccoaomt6l*


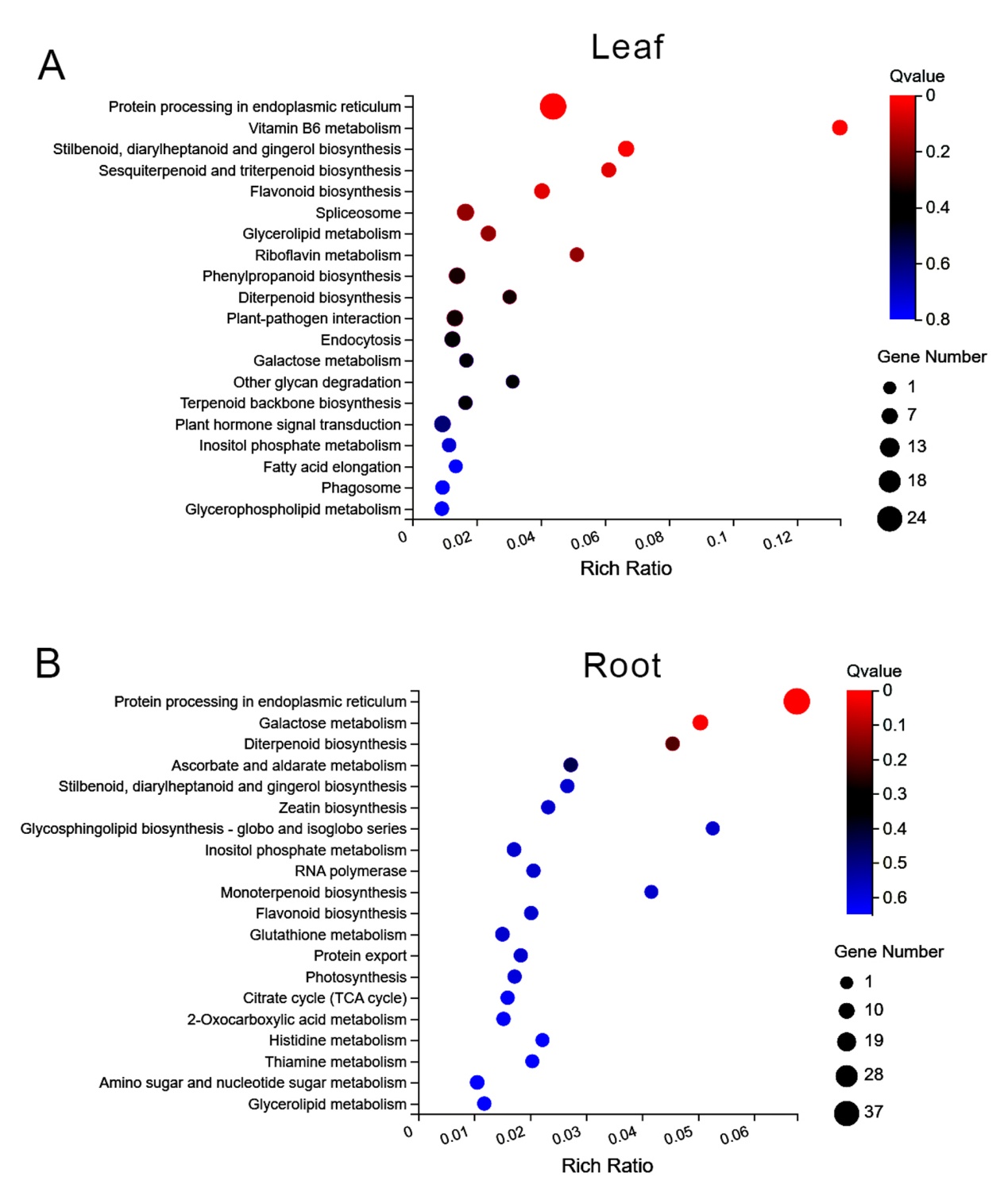


**Supplemental Figure 5.** KEGG classification of DEGs in leaves (A) and roots (B) between the wild-type and the double mutant *ccoaomt6 ccoaomt6l*
